# Fast dimension reduction and integrative clustering of multi-omics data using low-rank approximation: application to cancer molecular classification

**DOI:** 10.1186/s12864-015-2223-8

**Published:** 2015-12-01

**Authors:** Dingming Wu, Dongfang Wang, Michael Q. Zhang, Jin Gu

**Affiliations:** MOE Key Laboratory of Bioinformatics, TNLIST Bioinformatics Division & Center for Synthetic and Systems Biology, Department of Automation, Tsinghua University, Beijing, 100084 China; Department of Biological Sciences, Center for Systems Biology, University of Texas at Dallas, Richardson, TX 75080 USA

**Keywords:** Mutli-omics, Cancer, Low-rank approximation, Clustering, Dimension reduction, Algorithm

## Abstract

**Background:**

One major goal of large-scale cancer omics study is to identify molecular subtypes for more accurate cancer diagnoses and treatments. To deal with high-dimensional cancer multi-omics data, a promising strategy is to find an effective low-dimensional subspace of the original data and then cluster cancer samples in the reduced subspace. However, due to data-type diversity and big data volume, few methods can integrative and efficiently find the principal low-dimensional manifold of the high-dimensional cancer multi-omics data.

**Results:**

In this study, we proposed a novel low-rank approximation based integrative probabilistic model to fast find the shared principal subspace across multiple data types: the convexity of the low-rank regularized likelihood function of the probabilistic model ensures efficient and stable model fitting. Candidate molecular subtypes can be identified by unsupervised clustering hundreds of cancer samples in the reduced low-dimensional subspace. On testing datasets, our method LRAcluster (low-rank approximation based multi-omics data clustering) runs much faster with better clustering performances than the existing method. Then, we applied LRAcluster on large-scale cancer multi-omics data from TCGA. The pan-cancer analysis results show that the cancers of different tissue origins are generally grouped as independent clusters, except squamous-like carcinomas. While the single cancer type analysis suggests that the omics data have different subtyping abilities for different cancer types.

**Conclusions:**

LRAcluster is a very useful method for fast dimension reduction and unsupervised clustering of large-scale multi-omics data. LRAcluster is implemented in R and freely available via http://bioinfo.au.tsinghua.edu.cn/software/lracluster/.

**Electronic supplementary material:**

The online version of this article (doi:10.1186/s12864-015-2223-8) contains supplementary material, which is available to authorized users.

## Background

Cancer is a large family of lethal diseases which are killing millions of lives each year [1, 2]. Highly genetic heterogeneity makes it hard to develop general and effective treatments against cancer [3, 4]. One of the major goal of cancer multi-omics study is to discover possible cancer subtypes using molecule-level signatures, which can be used for more accurate diagnoses and treatments [5–8]. Several international collaborated projects, such as TCGA [9], ICGC [10], and CCLE [11] generated tons of cancer multi-omics data. However, we still face several challenges for analyzing such large-scale cancer multi-omics data: 1) need to handle different data types of different platforms at the same time, such as count based data of sequencing, continuous data of microarray and binary data of genetic variations; 2) the data dimension (the number of the molecular features) is much higher than the sample number; and 3) the big data volumes require efficient and robust computational algorithms.

The molecules involved in the same biological processes are usually highly correlated. It is commonly believed that the high-dimensional cancer genomic data can be reduced to a low-dimensional subspace associated to a few major biological processes [12–15], such as sustainable proliferation, apoptosis resistance, activated invasion and immune avoidance [16, 17]. Several efforts have been made to do such integration analysis [18–22]. To find the shared low-dimensional subspace across multiple data types, Shen et al. proposed a latent model iCluster + based on probabilistic principal component analysis, which used generalized linear models to transform continuous, discretized and count variables as a sparse linear regression on a set of latent driving factors. Then, cancer subtyping can be done in the reduced subspace consisting of the latent driving factors [21, 22]. Lock et al. proposed another Bayesian latent model (Bayesian consensus clustering, BCC) to simultaneously find the latent low-dimension subspaces and assign samples into different clusters [23]. However, the low computational efficiency limits its applications on large-scale cancer omics dataset.

In recent years, low-rank approximation (LRA) is becoming one kind of promising dimension reduction methods [20, 24]. In most cases, LRA is convex and can be solved using fast algorithm [25–27]. A few studies show the advantages of LRA for single data type analysis, such as cancer copy number variations [20, 28]. In this study, we formulated a novel low-rank approximation based integrative probabilistic model, which can deal with different data types with high computational efficiency and stability. It assumes that a few major biological factors determine a set of high-dimensional but low-rank systems parameters and the observed cancer omics data are generated based on these parameters. Results show that our method LRAcluster can run much faster than iCluster + with stable model fitting, which makes it possible to analyze large-scale cancer multi-omics data on a small server or even a personal computer.

Then, LRAcluster is applied on a large-scale TCGA multi-omics dataset of 11 different cancer types with four different data types, which is hard to be processed by previous methods. The pan-cancer analysis results suggest that different cancer types (or different tissue origins) can be generally grouped into independent clusters except squamous-like carcinomas in the reduced low-dimensional subspace. While, the single cancer type analysis results show that the multi-omics data have different subtyping capabilities for different cancer types.

## Methods

### LRAcluster overview

**Fig. 1:**
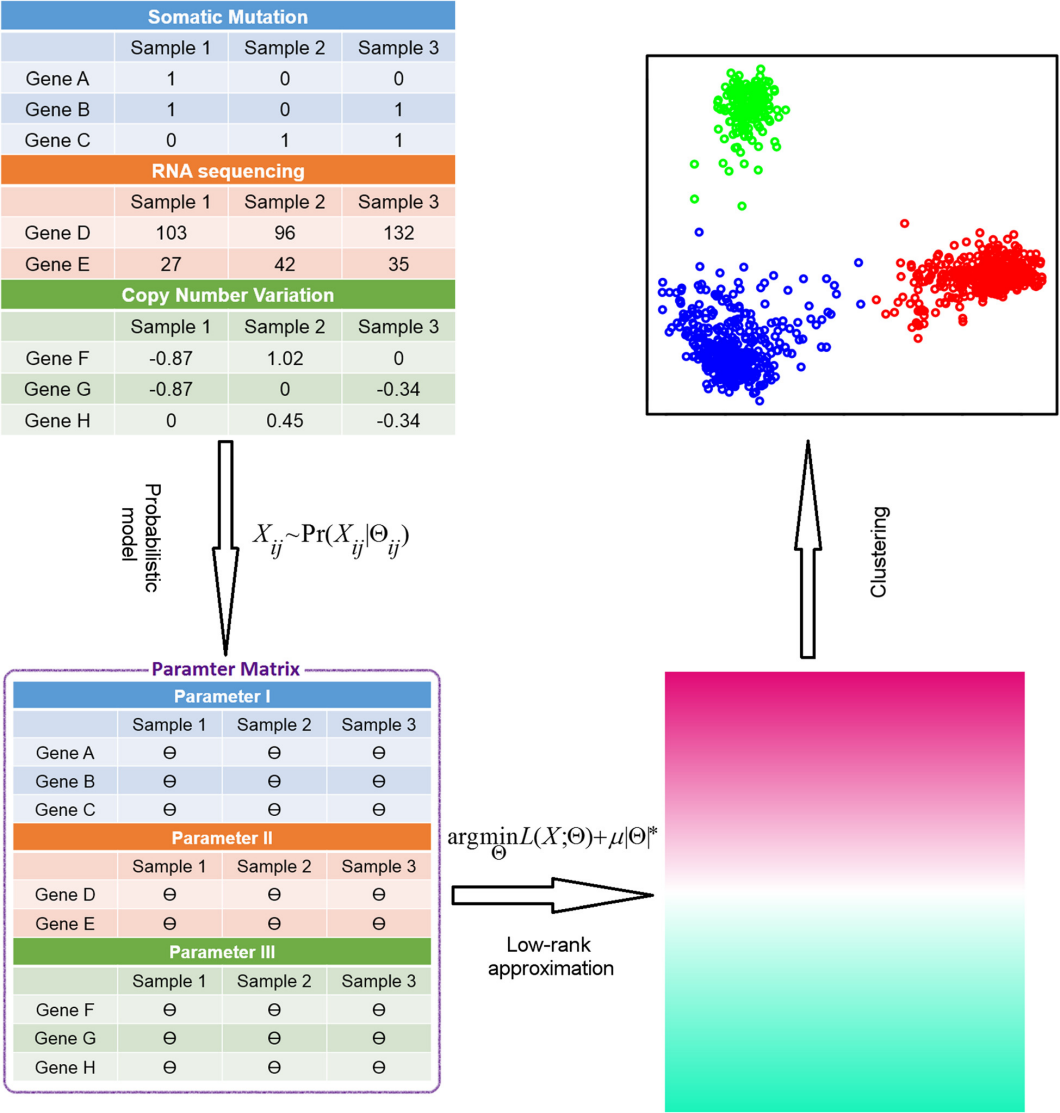
LRAcluster overview. LRAcluster receives 3 types (Gaussian, Poisson and Binary) of data as input. A probabilistic model with large amount of parameters are used to model the data. Low-rank approximation of the parameter matrix implies a latent subspace with low dimension. Clustering done on the reduced subspace generates the candidate molecular subtypes

LRAcluster is an unsupervised method to find the principal low-dimension subspace of large-scale and high-dimensional multi-omics data for molecular classification (Fig. 1). In LRAcluster model, the molecular features (such as somatic mutations, copy number variations, DNA methylations and gene expressions) are expressed as multiple observed data matrices. The probabilistic assumption is that each observed molecular feature of each sample is a random variable conditional on a hidden parameter. Thus, each observed data matrix is conditional on a size-matched parameter matrix and different types of data follow different probabilistic models (see below). The low-rank assumption of the parameter matrix leads to a penalty function corresponding to a structural complexity constraint of the model. Then, the low-rank parameter matrix can be decomposed into a low-dimensional representation of the original data, which will be used to identify candidate molecular subtypes.

### Probabilistic model

The *k*-th type of omics data are denoted as *X*_*ij*_^(*k*)^ (the row index represents the *i*-th molecular feature and the column index represent the *j*-th sample), while *Θ*^(*k*)^ denotes the size-matched parameter matrix of *X*^(*k*)^. The probabilistic model specifies the probability density (mass) function of the observations given the parameters for each data type as below:

- $\Pr \left({X}_{ij}^{(k)}\Big|{\varTheta}_{ij}^{(k)}\right)\propto \exp \left(-\frac{1}{2}{\left({X}_{ij}^{(k)}-{\varTheta}_{ij}^{(k)}\right)}^2\right)$ for real-type data, Gaussian distribution (CNV and DNA methylation data in this study);
- $\Pr \left({X}_{ij}^{(k)}\Big|{\varTheta}_{ij}^{(k)}\right)=\frac{e^{\varTheta_{ij}^{(k)}}}{1+{e}^{\varTheta_{ij}^{(k)}}}I\left({X}_{ij}^{(k)}=1\right)+\frac{1}{1+{e}^{\varTheta_{ij}^{(k)}}}I\left({X}_{ij}^{(k)}=0\right)$ for binary data, Bernoulli distribution (somatic mutation data in this study);
- $\Pr \left({X}_{ij}^{(k)}\Big|{\varTheta}_{ij}^{(k)}\right)\propto {\left({\lambda}_{ij}^{(k)}\right)}^{X_{ij}^{(k)}}{e}^{\left(-{\lambda}_{ij}^{(k)}\right)},\kern0.5em {\lambda}_{ij}^{(k)}={e}^{\varTheta_{ij}^{(k)}}$ for count data, Poisson distribution (RNAseq normalized count data in this study).

Categorical data can be transformed using dummy code and thus can be treated as binary variables.

The likelihood function of above probabilistic model is written as the minus log of the probability density (mass) function, which is:

1 $$ \mathrm{L}\left({\varTheta}^{(k)};{X}^{(k)}\right)=-{\displaystyle {\sum}_{ij}} \ln \left( \Pr \left({X}_{ij}^{(k)}\Big|{\varTheta}_{ij}^{(k)}\right)\right) $$

For integrative analysis, there are two or more observed data matrixes *X*^(*k*)^ (*k* = 1, 2, …, *K*). Thus the overall parameter matrix *Θ* stacks all the parameter matrices (*Θ*^(*k*)^) used for each observed data matrix. The overall likelihood function is the sum of the likelihood functions of different data types:

2 $$ \mathrm{L}\left(\varTheta \right)={\displaystyle {\sum}_k}\mathrm{L}\left({\varTheta}^{(k)};{X}^{(k)}\right) $$

The probabilistic model assumes that the observations *X*_*ij*_ are independently distributed conditional on the ultrahigh dimensional parameter matrix *Θ*. The prior assumption of the model is that *Θ* has low-rank structure. The low-rank assumption is used to penalize the freedom of the model and eventually leads to the following optimization problem:

3 $$ \arg \underset{\varTheta }{ \min }L\left(\varTheta \right)+\mu {\left|\varTheta \right|}^{*} $$

where *μ* is a tuning parameter and |•|* denotes the nuclear norm of the matrix [25].

### Fast low-rank approximation

The solution of the optimization problem (3) mimics a singular value thresholding method [26] which suggests a general framework to solve the optimization problem $\arg \underset{\varTheta }{ \min }f\left(\varTheta \right)+\mu {\left|\varTheta \right|}^{*}$ where *f* is a convex function. The iterative solution framework can be briefly expressed as the following steps:

1. initialize *Θ*^0^ and iterate the following two steps until convergence
2. $$ {\varTheta}^{2n+1}={\varTheta}^{2n}-{\delta}_n\nabla f $$
3. $$ {\varTheta}^{2n+2}={\mathrm{D}}_{\mu}\left({\varTheta}^{2n+1}\right) $$

∇*f* is the gradient of the un-regularized likelihood function (2) and *δ*_n_ is the step length. D_*μ*_ represents the “singular value shrinkage operator”: let us denote the singular value decomposition (SVD) of a matrix *Θ* as *Θ* = *UΣV*^*T*^, then D_*μ*_(*Θ*) = *U*D_*μ*_(*Σ*)*V*^T^. D_*μ*_(*Σ*) is a diagonal matrix with the same size as *Σ* and each diagonal element is the shrinkage of the singular value of *Σ*. For a positive singular value *λ*, the shrinkage result is (*λ*–*μ*) when *λ* > *μ* and 0 when *λ* ≤ *μ*.

The objective function of LRAcluster is convex, so any initial value of the iteration will converge to the global minimum. LRAcluster simply initializes *Θ* as a zero matrix. The original framework needs a user defined constraint parameter *μ* which is hard to choose in practical use. Instead of *μ*, LRAcluster receives the rank *r* (also the target dimension) as the user defined constraint parameter. *μ* is automatically chosen as the rank *r* + 1 largest singular value in each iteration. The choice of *μ* is to guarantee that *Θ* has rank *r* and the shrinkage has minimal effect on *Θ*. For simple “matrix completion problem”, Cai et al. proves that when the step length *δ* is between 0.5 and 2, the algorithm converges definitely [26]. LRAcluster set *δ* as 0.5, which ensures convergence for real applications in this study.

The target rank (or dimension) *r* is the only user-defined parameter in dimension reduction step. The log likelihood − L(*θ*; *X*) corresponding to the optimized solution θ* (denoted as ℒ_r_^*^) is used for guiding the choice of parameter *r*: for the same dataset, larger *r* means weaker penalization of the model freedom and leads to better data fitting (larger likelihood ℒ_r_^*^). Thus, ℒ_*r* = 0_^*^ is the minimum and ℒ_*r* = + ∞_^*^ is the maximum among all the ℒ_r_^*^. The quantity ℒ_r_^*^ describes to what extend the model fits the data. As LRAcluser mainly deals with large dataset, ℒ_r_^*^ is usually a big value. So, instead of ℒ_r_^*^, LRAcluster uses the normalized quantity $\frac{{\mathrm{\mathcal{L}}}_{r=+\infty}^{*}-{\mathrm{\mathcal{L}}}_r^{*}}{{\mathrm{\mathcal{L}}}_{r=+\infty}^{*}-{\mathrm{\mathcal{L}}}_{r=0}^{*}}$ (between 0 and 1) as “explained variation” for choosing a desirable rank *r*. We will describe the basic principles for the choice of rank *r* in Results section.

### Dimension reduction and clustering

The dimension reduction is straightforward after getting the low-rank matrix *Θ*. As the rank of *Θ* is no more than *r*, the singular vector decomposition (SVD) of that matrix *Θ* = *UΣV*^T^ has *Σ* with no more than *r* non-zero singular values. Thus the first *r* columns of *ΣV*^T^ are just the dimension reduction result of the original data matrix *X* with the target dimension (rank) *r*.

LRAcluster uses *k*-means to identify the candidate molecular subtypes in the reduced low-dimensional subspace. Silhouette values [29] is used to determine the cluster number *k*. Any other unsupervised clustering algorithm can be used instead of *k*-means.

### Datasets

In this study, all the datasets were downloaded from publicly released TCGA level 3 data (processed data from UCSC Cancer Genome Browser [30]). No ethics approval is required for this study. The whole dataset consists of 11 types of cancer (BRCA, COAD, GBM, HNSC, KIRC, LGG, LUAD, LUSC, PRAD, STAD, and THCA) with somatic mutations, copy number variations, DNA methylations and gene expressions. For somatic mutation and copy number variation data, our preliminary studies indicate that the massive passenger variations of the complete datasets deteriorated the clustering stability. Thus, only the somatic mutations and copy number variations of the ~500 genes reported as “causally implicated in cancer” in COSMIC [31] were included in this study. For DNA methylation data using Illumina HumanMethylation450 BeadChip (450 k array), probes annotated as “promoter_associated” (based on the annotations of IlluminaHumanMethylation450k.db [32]) were selected (if a gene has multiple promoter associated probes, only one of them was chosen). Overall, ~8,000 probes were used. The normalized count-based data from RNA-Seq were all included with ~20,000 genes.

The three cancer-type testing dataset consists of BRCA, COAD, LUAD cancer types with RNA-Seq and and 450 k DNA methylation data. The other datasets consists of all the four data types described as above.

## Results

LRAcluster is a computational-efficient method for fast dimension reduction and integrative clustering of large-scale cancer multi-omics data. We first show the performances and parameter tuning of LRAcluster on a three cancer-type testing dataset and a breast cancer dataset labeled with ER+/ER- subtypes. Then, it was applied on the large-scale TCGA pan-cancer dataset.

### The computational performances of LRAcluster

A three cancer-type dataset was used to compare the clustering performances and time consumption between LRAcluster and iCluster+. The molecular features (genes for expression data and probes for DNA methylation data) with largest variances across all samples are selected to construct datasets of different sizes. The smallest dataset containing top 100 molecular features of each data type is used to test LRAcluster and iCluster+’s clustering performances with different target dimension (from 2 to 10). Time consumption of the two methods was recorded for datasets with different feature sizes (from 100 to 5000 features). iCluster + runs under both normal model (random initialization of penalty parameter for better model) and simple model (fixed penalty parameter).

**Fig. 2:**
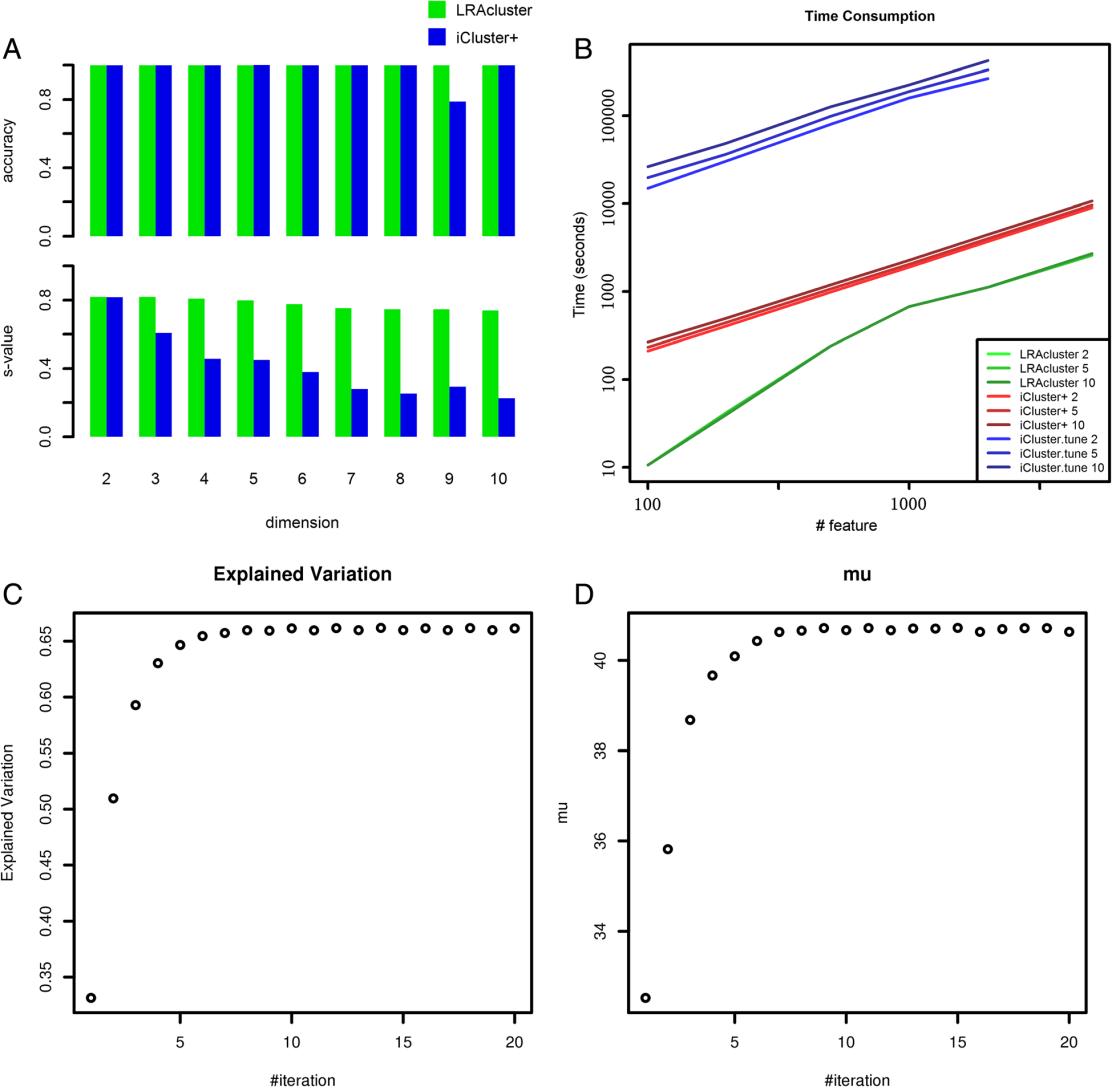
Performance of LRAcluster. **a** the classification accuracy and silhouette value against the dimension of the reduced subspace (the cluster number is set as three) on the three cancer-type testing dataset. **b** Time consumption of LRAcluster and iCluster+. The number behind the method’s name is the dimension of the latent subspace. iCluster + represents the method that do not tune the penalty parameter. iCluster.tune represents the method tuning the penalty parameter. **c** and **d** the dynamic changes of the explained variance and penalty parameter *μ* as the algorithm iterates

We found that both LRAcluster and iCluster + got high classification accuracy for the three cancer types in the reduced low-dimension subspaces (Fig. 2a). The only exception is for iCluster + with target dimension 9: it misclassified COAD and LUAD samples, which may be caused by unstable model fitting of iCluster+. But, the silhouette values show that the clustering performances of LRAcluster are superior to iCluster+, especially when the target dimension is large. These results indicate that iCluster + will encounter local optimal problems when the model becomes complex, while the convexity of LRAcluster model ensures stable model fitting (Fig. 2a). For the time cost, LRAcluster runs ~5 fold faster than iCluster + with fixed penalty parameter and much faster (~300 fold) if that parameter is optimized (the programs are all running under single thread model) (Fig. 2b).

**Fig. 3:**
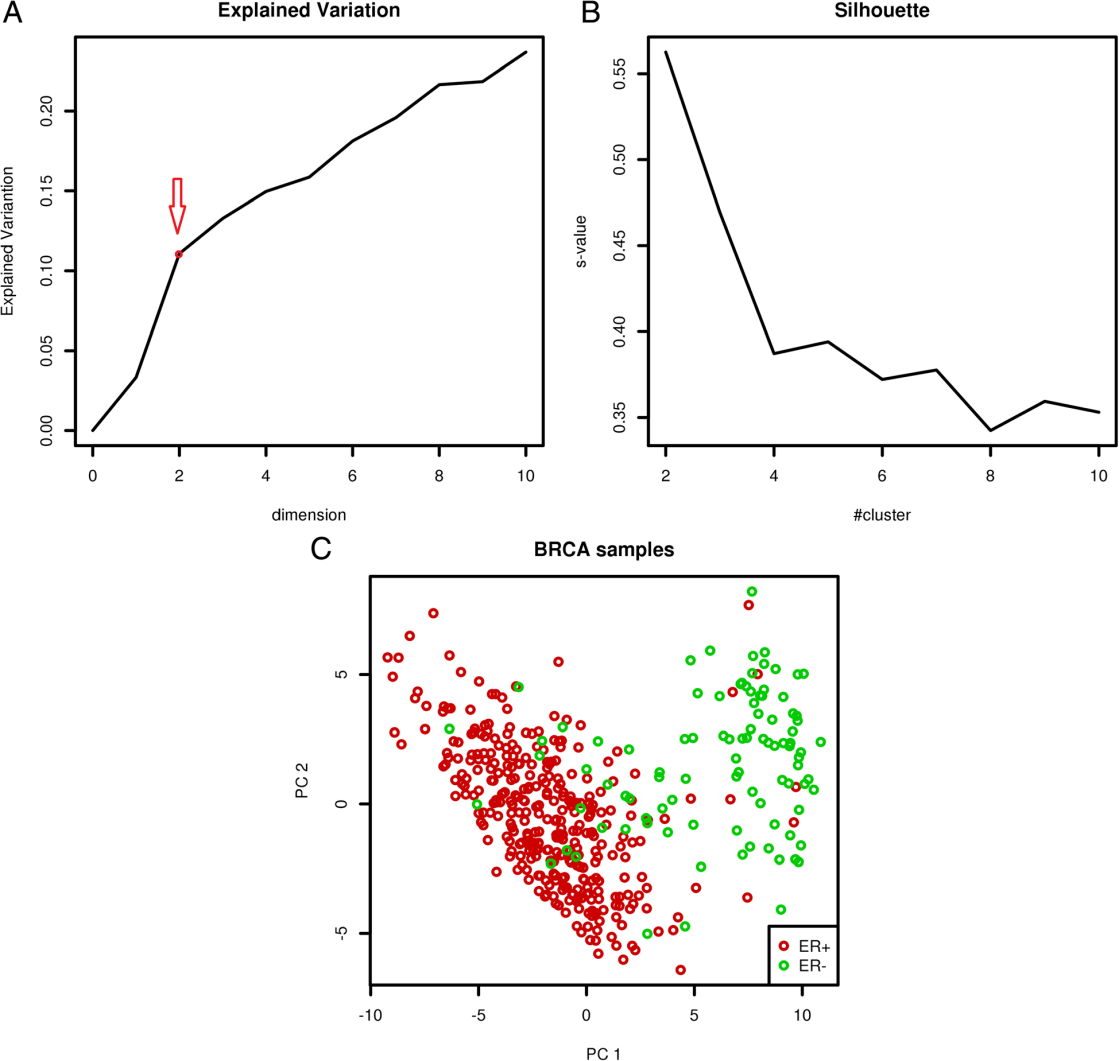
The curves for parameter choice. **a** the curve of “explained variance” against dimension. **b** the curve of silhouette value against cluster number. **c** the scatter plot of BRCA samples in the reduced 2-dimensional subspace

The convergence is an important issue for model fitting. The dynamic changes of the “explained variance” and the penalty parameter *μ* demonstrated that LRAcluster can quickly converge within only a few iterations (Fig. 2c & d). There are two important parameters in LRAcluster: the rank (or dimension) of the reduced subspace *r* and the cluster number *c*. To illustrate how to choose these parameters empirically, we used the BRCA dataset with known ER+/ER- subtypes as an example: the dimension *r* can be chosed according to the curve of “explained variance” (Fig. 3a) and the cluster number *c* can be chosen according to the curve of silhouette value (s-value) (Fig. 3b). For the BRCA dataset, dimension *r* should be chosen as 2, because there was a turning point at 2 on the curve of the “explained variance” (Fig. 3a). This empirical rule is based on the principle that the increase of model fitness is much slower after the changing point. The choice of cluster number *c* is straightforward: larger s-value indicates better clustering performance. For the BRCA dataset, the largest s-value was achieved when *c* = 2 (Fig. 3b). Results show that LRAcluster can find two subtypes in the reduced 2-dimensional subspace and the identified subtypes are highly consistent with known ER+/ER- subtypes (accuracy 92.1 %) (Fig. 3c).

### Application on the large-scale TCGA pan-cancer dataset

By applying LRAcluster on the TCGA pan-cancer dataset (11 different cancer types, 3,319 samples and four different data types including somatic mutations, copy number variations, DNA methylations, and gene expressions), we get ten clusters in the reduced ten-dimension subspace (Table 1). The dimension and the cluster number were determined according to the curves of “explained variances” and s-values, respectively, as above principles (curves are shown in Additional file 1: Figure S1 & S2).

**Table 1:** The unsupervised clustering results of pan-cancer analysis

	BRCA	COAD	GBM	HNSC	KIRC	LGG	LUAD	LUSC	PRAD	STAD	THCA	Total
C1	1	0	0	286	0	0	0	6	0	0	0	293
C2	0	0	0	0	0	1	0	0	0	0	411	412
C3	0	0	41	0	0	451	0	0	0	0	0	492
C4	0	0	0	0	0	0	0	0	0	231	0	231
C5	0	0	0	0	0	0	0	0	293	0	0	293
C6	0	190	0	1	0	0	2	0	1	0	0	194
C7	3	17	0	0	1	0	406	7	0	0	3	437
C8	0	0	0	0	240	0	0	0	0	0	0	240
C9	448	0	1	2	1	0	4	1	0	0	0	457
C10	8	1	0	195	0	0	6	60	0	0	0	270
Total	460	208	42	484	242	452	418	74	294	231	414	3319

Results show that most samples from the same cancer types are grouped as independent clusters. These results are similar with a recent pan-cancer study [8]. The two brain cancers (LGG and GBM) are grouped together (Cluster C3). Only HNSC are separated into two major clusters (Cluster C1 & C10) and the samples (40.3 % of HNSC) in Cluster C10 are clustered together with LUSC samples (81.1 % of LUSC), which indicates that the squamous carcinomas of different tissue origins may share some common molecular mechanisms. A recent work also reported an integrative network-based stratification (jNBS) pan-cancer clustering analysis on TCGA dataset, which incorporated multi-omics data with the information of a pre-given gene network [33]. Generally speaking, it reported similar results with LRAcluster: most of cancer types are separately clustered according to their tissue origin, and two types of squamous carcinomas, head/neck squamous carcinoma and lung squamous carcinoma are cluster together. But it found more cross tissue type clusters. Because the jNBS analysis only used genetic (mutation & CNV) and epigenetic (DNA methylation) data, the results are hard to be directly compared. The molecular signatures associated with the pan-cancer clusters were shown in Additional file 1: Figure S3.

**Fig. 4:**
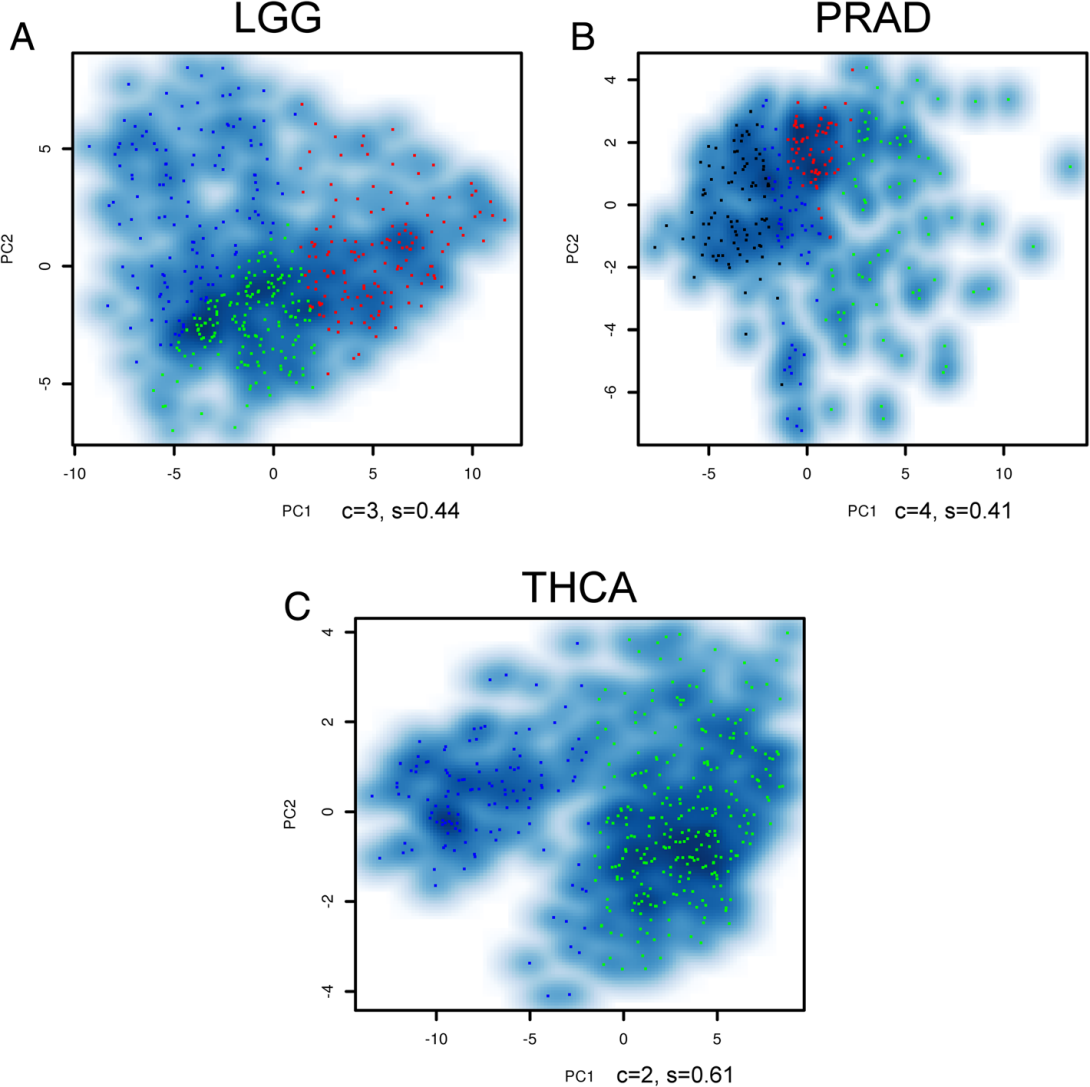
The molecular subtypes identified by LRAcluster. (**a**) is for LGG, (**b**) for PRAD and (**c**) for THCA. The scatter plots show all the samples in the corresponding reduced 2-dimensional subspace. Different colors represent different molecular subtypes identified by LRAcluster, *c* indicates the number of identified clusters and *s* shows the silhouette value

Then, LRAcluster was applied on the 11 cancer types separately. The results suggest that the omics data have different subtyping abilities of different cancer types (Table 2). BRCA, LGG, PRAD, and THCA datasets get high silhouette values. As described above, the BRCA subtypes are significantly associated with ER status. But, there are no significant differences of overall survival among the identified molecular subtypes in LGG, PRAD, and THCA (the scatter plots of the samples in reduced subspace were shown in Fig. 4). For the remaining 7 cancer types, LRAcluster did not find strong molecular subtypes based on current omics data.

**Table 2:** The results of single-cancer analysis

Cancer	Dimension^a^	#Cluster^b^	Silhouette values
BRCA	2	2	0.55
COAD	4	4	0.40
GBM	8	2	0.35
HNSC	7	3	0.26
KIRC	6	2	0.36
LGG	2	3	0.44
LUAD	5	2	0.34
LUSC	5	4	0.32
PRAD	2	4	0.41
STAD	4	3	0.37
THCA	2	2	0.61

^a^The dimension of the reduced space is determined according to the curve of the explained variations of each cancer type

^b^The number of clusters is determined according to the curve of the within cluster variances

## Conclusion

LRAcluster probabilistically models the observed data conditional on the size-matched parameters. The low-rank constraint is the key to get the low-dimensional representation of the original data. And the convexity of the regularized likelihood function provides efficient gradient-descent algorithm for model fitting. Results show that LRAcluster runs fast with high classification accuracy and it is suitable for large-scale cancer multi-omics analysis.

## Discussions

In LRAcluster probabilistic model, the real-type data are modeled as Gaussian-distributed random variables with variance 1. Though the assumption of all features having the same variance seems unnatural for any dataset as the different features should have different variance, it is consistent with the simple method of principle component analysis. Minus log likelihood function of the real-type data is $\frac{1}{2}{\left({X}_{ij}-{\varTheta}_{ij}\right)}^2$ which is the same as the loss function of principle component analysis (PCA). So, if there are only real-type data as input, the LRAcluster solution is in principle the same as the PCA. The only difference is the scale of each principle component because the LRAcluster considers the L_1_ norm but PCA considers the L_0_ norm.

LRAcluster receives the rank *r* of the matrix *Θ* as the user-defined parameter instead of the original parameter *μ*. This setting makes the dimension reduction more straightforward: *r* is just the target dimension of the reduced subspace. From computational view, *μ* and *r* have the same function as they are both used to penalize the complexity of the probabilistic model.

LRAcluster does not penalize the association between molecular features and the reduced subspace (latent factors) via sparsity assumption. It is a better strategy to find the molecular features associated the identified clusters or subtypes by molecular signature analysis: find the significantly differential features between the samples in that cluster and all the other samples (please see the heatmap of the selected molecular features of TCGA pan-cancer analysis in Additional file 1: Figure S3). Besides, LRAcluster will prefer the inter-omics features with large co-variances implied by the low-rank assumption (for example, the significantly correlated CNVs and mRNA expressions). The inter-omics regulatory information can be modeled as a separate pre-processing step to find the cancer driving factors and then only the molecular features significantly associated with these drivers are used as the input of LRAcluster.

Joint non-negative matrix factorization (jNMF) is another strategy to find the shared principal subspace across multiple omics datasets [34, 35]. Theoretically, NMF can be treated as a matrix version of latent factor analysis. jNMF will also encounter the optimization difficulty of non-convey loss function. But the advantage of jNMF is that the model can also get the molecular features (or called as modules) significantly associated each dimension.

## Additional file

Additional file 1:
**This file contains Supplementary Figures S1-S3.** **Figure S1.** The curve of “explained variance” against the target rank *r*. **Figure S2.** The curve of silhouette value against cluster number. **Figure S3.** Heatmap of the molecular signatures associated with the identified clusters of the TCGA pan-cancer dataset. (DOCX 2330 kb)

## References

[1] Ferlay J, Soerjomataram I, Dikshit R, Eser S, Mathers C, Rebelo M (2015). Cancer incidence and mortality worldwide: sources, methods and major patterns in GLOBOCAN 2012. Int J Cancer.

[2] Siegel R, Ma J, Zou Z, Jemal A (2014). Cancer statistics, 2014. CA Cancer J Clin.

[3] Bedard PL, Hansen AR, Ratain MJ, Siu LL (2013). Tumour heterogeneity in the clinic. Nature.

[4] Burrell RA, McGranahan N, Bartek J, Swanton C (2013). The causes and consequences of genetic heterogeneity in cancer evolution. Nature.

[5] Hayhoe FG (1988). Classification of acute leukaemias. Blood Rev.

[6] Yan H, Peng Z-G, Wu Y-L, Jiang Y, Yu Y, Huang Y (2005). Hypoxia-simulating agents and selective stimulation of arsenic trioxide-induced growth arrest and cell differentiation in acute promyelocytic leukemic cells. Haematologica.

[7] Yersal O, Barutca S (2014). Biological subtypes of breast cancer: Prognostic and therapeutic implications. World J Clin Oncol.

[8] Hoadley KA, Yau C, Wolf DM, Cherniack AD, Tamborero D, Ng S (2014). Multiplatform analysis of 12 cancer types reveals molecular classification within and across tissues of origin. Cell.

[9] The Cancer Genome Atlas [http://cancergenome.nih.gov/]

[10] Hudson TJ, Anderson W, Artez A, Barker AD, Bell C, Bernabe RR (2010). International network of cancer genome projects. Nature.

[11] Barretina J, Caponigro G, Stransky N, Venkatesan K, Margolin AA, Kim S (2012). The Cancer Cell Line Encyclopedia enables predictive modelling of anticancer drug sensitivity. Nature.

[12] Huang E, Ishida S, Pittman J, Dressman H, Bild A, Kloos M (2003). Gene expression phenotypic models that predict the activity of oncogenic pathways. Nat Genet.

[13] Li L, Li H (2004). Dimension reduction methods for microarrays with application to censored survival data. Bioinforma Oxf Engl.

[14] Li H, Gui J (2004). Partial Cox regression analysis for high-dimensional microarray gene expression data. Bioinforma Oxf Engl.

[15] Jia P, Pao W, Zhao Z (2014). Patterns and processes of somatic mutations in nine major cancers. BMC Med Genomics.

[16] Hanahan D, Weinberg RA (2000). The hallmarks of cancer. Cell.

[17] Hanahan D, Weinberg RA (2011). Hallmarks of cancer: the next generation. Cell.

[18] Alter O, Golub GH (2004). Integrative analysis of genome-scale data by using pseudoinverse projection predicts novel correlation between DNA replication and RNA transcription. Proc Natl Acad Sci U S A.

[19] Yuan Y, Savage RS, Markowetz F (2011). Patient-specific data fusion defines prognostic cancer subtypes. PLoS Comput Biol.

[20] Lock EF, Hoadley KA, Marron JS, Nobel AB (2013). Joint and individual variation explained (JIVE) for integrated analysis of multiple data types. Ann Appl Stat.

[21] Mo Q, Wang S, Seshan VE, Olshen AB, Schultz N, Sander C (2013). Pattern discovery and cancer gene identification in integrated cancer genomic data. Proc Natl Acad Sci U S A.

[22] Shen R, Olshen AB, Ladanyi M (2009). Integrative clustering of multiple genomic data types using a joint latent variable model with application to breast and lung cancer subtype analysis. Bioinforma Oxf Engl.

[23] Lock EF, Dunson DB (2013). Bayesian consensus clustering. Bioinforma Oxf Engl.

[24] Yuan M, Ekici A, Lu Z, Monteiro R (2007). Dimension reduction and coefficient estimation in multivariate linear regression. J R Stat Soc Ser B-Stat Methodol.

[25] Candes EJ, Recht B (2009). Exact Matrix Completion via Convex Optimization. Found Comput Math.

[26] Cai JF, Candès EJ, Shen Z (2010). A singular value thresholding algorithm for matrix completion. SIAM J Optim.

[27] Hsieh CJ, Olsen PA. Nuclear Norm Minimization via Active Subspace Selection. *Proc 31st Int Conf Mach Learn.* 2014.

[28] Zhou X, Liu J, Wan X, Yu W (2014). Piecewise-constant and low-rank approximation for identification of recurrent copy number variations. Bioinforma Oxf Engl.

[29] Rousseeuw P (1987). silhouettes - A graphical aid to the integration of cluster-analysis. J Comput Appl Math.

[30] Goldman M, Craft B, Swatloski T, Cline M, Morozova O, Diekhans M (2015). The UCSC Cancer Genomics Browser: update 2015. Nucleic Acids Res.

[31] Forbes SA, Beare D, Gunasekaran P, Leung K, Bindal N, Boutselakis H (2015). COSMIC: exploring the world's knowledge of somatic mutations in human cancer. Nucleic Acids Res.

[32] Triche T, Jr. IlluminaHumanMethylation450k.db: Illumina Human Methylation 450k annotation data.

[33] Liu Z, Zhang S (2015). Tumor characterization and stratification by integrated molecular profiles reveals essential pan-cancer features. BMC Genomics.

[34] Zhang S, Li Q, Liu J, Zhou XJ (2011). A novel computational framework for simultaneous integration of multiple types of genomic data to identify microRNA-gene regulatory modules. Bioinforma Oxf Engl.

[35] Zhang S, Liu C-C, Li W, Shen H, Laird PW, Zhou XJ (2012). Discovery of multi-dimensional modules by integrative analysis of cancer genomic data. Nucleic Acids Res.

