# Supplementary material for: Fast dimension reduction and integrative clustering of multi-omics data using low-rank approximation: application to cancer molecular classification
Source: BMC Genomics. 2015 Dec 1;16:1022. doi: 10.1186/s12864-015-2223-8 (PMC4667498; doi:10.1186/s12864-015-2223-8)
Supplement: Additional file 1: — This file contains Supplementary Figures S1-S3. Figure S1. The curve of “explained variance” against the target rank r. Figure S2. The curve of silhouette value against cluster number. Figure S3. Heatmap of the molecular signatures associated with the identified clusters of the TCGA pan-cancer dataset. (DOCX 2330 kb) [file 12864_2015_2223_MOESM1_ESM.docx]

# Supplementary Figures


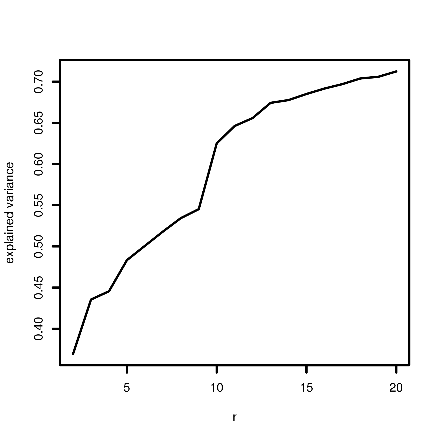


**Figure S1**. “Explained variance” against the parameter $r$.


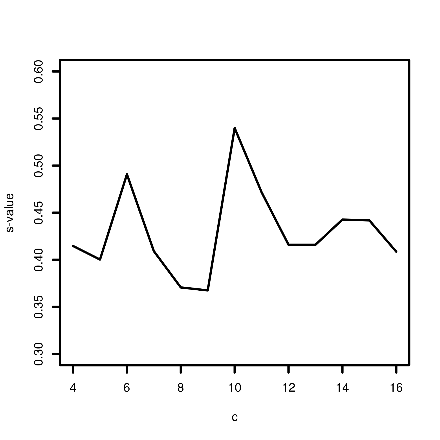


**Figure S2**. Silhouette value against cluster number.

Application of LRAcluster on TCGA pan-cancer data shows 10 clusters among 11 types of cancer. Based on these 10 clusters, we find individual genes having the largest ratio of between-cluster dividing by variance within-cluster variance (the criterion used by fisher linear discriminant analysis). Top 100 genes of somatic mutation, copy number variation and top 500 genes of DNA methylation, gene expression are used to construct the “signature” of these 10 clusters. The heatmap is shown in Figure S3.

**
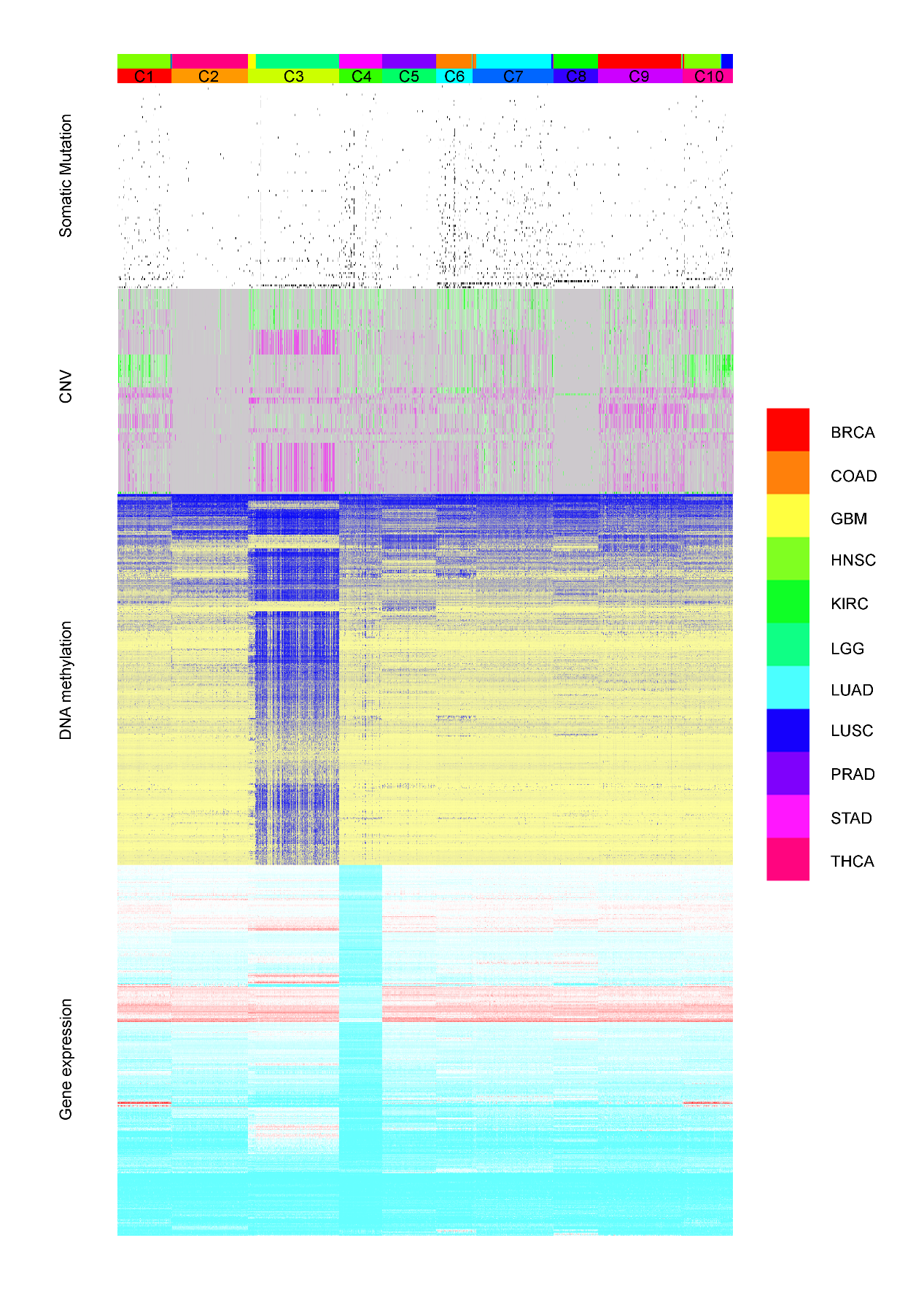
Figure S3**. Heatmap of the molecular signatures associated with the identified clusters from the TCGA pan-cancer dataset. Top color bars indicate the known cancer types and the clusters identified by LRAcluster.
